# Application of the Immunoscore as prognostic tool for hepatocellular carcinoma

**DOI:** 10.1186/s40425-016-0182-5

**Published:** 2016-11-15

**Authors:** Annacarmen Petrizzo, Luigi Buonaguro

**Affiliations:** Laboratory of MolecularBiology and ViralOncology, Istituto Nazionale per lo Studio e la Cura dei Tumori, “Fondazione Pascale” - IRCCS, Via Mariano Semmola, 1, 80131 Naples, Italy

**Keywords:** Hepatocellular carcinoma, TNM Stage, Immunoscore, Tumor-infiltrating lymphocytes, Programmed Death Ligand 1 (PD-L1)

## Abstract

To date, the American Joint Committee on Cancer (AJCC) and the Union for International Cancer Control (UICC) tumor, nodes, metastasis (TNM) classification represents the standard system for evaluation of prognosis in solid tumors. However, the clinical outcome can be significantly different in patients with the same TNM stage. Therefore, many efforts have been made aiming to define new prognostic parameters.

Indeed, analyses conducted in large cohorts of colorectal cancer patients emphasized the prognostic value of tumor-infiltrating lymphocytes, leading to the development of a prognostic score referred to as “Immunoscore”.

In this commentary, we recapitulate the study by Gabrielson and colleagues, recently published in Cancer Immunology Research, addressing the role of intratumoral CD3^+^ and CD8^+^ T cells as well as as prognostic markers for hepatocellular carcinoma.

The authors demonstrate that Immunoscore represents a valuable prognostic marker in patients with hepatocellular carcinoma who have undergone primary tumor resection, supporting its application in a tumor setting other than colorectal cancer.

## Background

Hepatocellular carcinoma (HCC) is the most common primary liver malignancy and accounts for about 6 % of all new cancers diagnosed worldwide. It is the third and the fifth leading cause of death from cancer in men and women, respectively. More than 50 % of HCC cases can be attributed to HBV chronic infection, whereas HCV chronic infection accounts for 30 % of cases. Approximately 15 % of HCC cases can be associated with non-viral causes, including alcohol, aflatoxins, metabolic liver diseases, steatosis, non-alcoholic fatty liver disease. The overall prognosis for HCC patients is poor, with a 5-year survival rate of 5–6 % [1, 2].

Several strategies are employed in the management of HCC according to the extent of liver disease. In particular, in early-stage HCC, surgery (i.e., tumor resection and liver transplantation) represents the standard method. However, radiofrequency (RF), thermal ablation and trans-arterial chemoembolization (TACE) provide a second line therapy for patients with unresectable HCC or for those who are not eligible for liver transplantation [3].

In this perspective, evaluation of prognosis represents a crucial step for proper management of HCC patients. Accordingly, HCC prognosis is closely related to its stage. To date, several staging systems are employed to estimate life expectancy of HCC patients, none of which has been universally adopted. In particular, four features have been recognized as being important determinants of survival: the severity of underlying liver disease, the size of the tumor, extension of the tumor into adjacent structures, and the presence of metastases [4, 5]. The TNM classification system, uses T (i.e., tumor size and number), N (i.e., regional lymph node involvement) and M (i.e., metastasis) parameters to stage the disease and stratify patients according to the tumor characteristics (Table 1) [6].

**Table 1:** TNM classification for staging of hepatocellular carcinoma

Stage	T	N	M
I	T1	N0	M0
II	T2	N0	M0
IIIa	T3a	N0	M0
IIIb	T3b	N0	M0
IIIc	T4	N0	M0
IVa	Any T	N1	M0
IVb	Any T	Any N	M1

T1: single tumor without vascular invasion

T2: single tumor with vascular invasion or multiple tumors, none > 5 cm

T3a: multiple tumors > 5 cm

T3b: tumor involving a major branch of the portal or hepatic vein

T4: tumor with direct invasion of adjacent organs other than gallbladder or with perforation of visceral peritoneum

N0: no regional lymph node metastasis

N1: regionallymph node metastasis

M0: no distant metastasis

M1: distant metastasis

However, the clinical outcome (i.e., relapse-free survival (RFS) and overall survival (OS)) can be significantly different in HCC patients within the same TNM stage of disease. Therefore, many efforts have been made to define new parameters with more precise prognostic value and the search for HCC prognostic markers, in a setting of extreme heterogeneity, is gaining momentum. Several biomarkers have been described, so far, for both biological characterization of the tumor and evaluation of prognosis.

In particular, the prognostic significance of the estrogen receptor (ER) in patients with HCC was investigated in a study showing that patients with wild-type ER may experience better survival than those presenting a variant ER [7]. However, ER characterization requires quite invasive procedure (i.e., liver biopsy). In this perspective, more recently, several studies have been focused on the identification of new serological markers for routine analysis application (reviewed in [8, 9]).

Serum Dickkopf-1 (DKK1) has been suggested as a potential prognostic marker for HCC in combination with alpha-fetoprotein (AFP) [10]. Similarly, a recent study showed that plasma osteopontin (OPN) combined with serum AFP can be used as prognostic marker in patients with early-stage HCC [11]. In addition, low serum levels of vascular endothelial growth factor (VEGF) seem to be associated with longer survival [12]. Moreover, plasma levels of insulin-like growth factor-1 (IGF-1) correlate with time-to-recurrence, as well as OS [13]. Although these new serological markers have shown promising results, they still require further evaluation and clinical validation.

Interestingly, the HCC microenvironment comprises a network of cells that play a critical role in tumor progression influencing prognosis. Several studies have shown a correlation between HCC prognosis and tumor-infiltrating cells affecting tumor growth, invasion, angiogenesis and metastasis, including: tumor associated macrophages (TAMs), hepatic stellate cells (HSCs), cancer-associated fibroblasts (CAFs), neutrophils, cancer stem-like cells (CSLCs) and Tregs. Unfortunately, none of these cells is validated yet for routine prognostic assessment [14].

In this scenario, the pioneering study of Galon’s group conducted on a large cohort of colorectal cancer (CRC) patients which led to the assessment of the tumor-infiltrating immune cells as valuable prognostic marker for the treatment of CRC [15]. The type, density and location of immune cells within distinct tumor regions, including tumor interior (TI) and invasive margin (IM), referred to as “Immunoscore”, was recognized as better predictor of clinical outcome than the standard TNM stage classification [16, 17].

### Tumor-infiltrating lymphocytes (TILs) as prognostic factor for HCC

In line with such evidence, the study by Gabrielson et al. recently published in Cancer Immunology Research represents one of the first papers addressing the cumulative role of intratumoral CD3^+^ and CD8^+^ T cells, as well as programmed death ligand 1 (PD-L1) as prognostic markers for hepatocellular carcinoma [18].

The authors reviewed survival data of 65 HCC patients (stage I to IV), who underwent primary tumor resection between 2006 and 2015. The mean follow-up was 39.7 months. Surgical tissue specimens were analyzed. Immunohistochemistry (IHC) staining with monoclonal antibodies to CD3, CD8 and PD-L1 was performed for biomarker imaging in TI, IM and noncancerous liver parenchyma. The median immune cell density was used to stratify patients into groups according to the Immunoscore as defined by Galon et al. in CRC [17].

Patients with low densities of CD3^+^ and CD8^+ ^T cells in both TI and IM tumor regions were classified as Im0; patients with one high density for one marker were classified as Im1; patients with two, three and four high densities for the two markers were classified as Im2, Im3, and Im4, respectively.

The authors observed a statistically significant association between intratumoral CD3^+^ and CD8^+^ T cells and frequency of HCC recurrence. In particular, patients with a high density of CD3^+^ immune infiltrates in the TI and IM regions experienced recurrence of HCC only in 15 % of cases compared with 44 % in those with a low CD3^+^ cell density (*P* = 0.027). Similarly, patients with a high density of CD8^+^ immune infiltrates experienced recurrence of HCC in 15 % of cases compared with 45 % of those with a low CD8^+^ T cell density (*P* = 0.014). The frequency of HCC recurrence in each Immunoscore subgroup was 65 % for Im0, 22 % for Im1, 10 % for Im2, 10 % for Im3, and 11 % for Im4. In addition, high densities of CD3^+^ and CD8^+^ T cells in both TI and IM regions, along with corresponding Immunoscores, were significantly associated with a prolonged RFS (*P* = 0.002). Interestingly, the present study confirms the data of a previous study by Sun and colleagues who showed that distribution and densities of CD3^+^ and CD8^+^ T cells in central tumor regions represent a predictive tool for HCC progression [19].

The authors also tested PD-L1 expression in relation to CD3^+^ and CD8^+^ T cells density. Indeed, expression of PD-L1 correlated with high density of CD3^+^ and CD8^+^ T cells (*P* = 0.024 and 0.005, respectively). PD-L1 expression predicted lower recurrence rate (*P* = 0.034), as well as prolonged RFS (*P* = 0.029) [18].

Taken together, these data underline the relevance of Immunoscore and PD-L1 expression as prognostic markers in patients who have undergone HCC resection.

### Perspectives

The study by Gabrielson et al. clearly pointed out a positive correlation between PD-L1 expression and CD3^+^ and CD8^+^ T cells densities. Interestingly, 19 samples showed PD-L1 expression in non-malignant cells around the tumor area. The authors argued that PD-L1 inhibitory pathway represents a negative feedback mechanism that follows, rather than precedes, CD8^+^ T cell infiltration [18]. The authors also described mechanistic studies showing that upregulated expression of PD-L1 in mice is driven by IFNγ and depends on the presence of CD8^+^ T cells in the tumor microenvironment.

Several studies have been focused on analysis of the prognostic significance of PD-L1 expression with very contrasting results [20–22].

However, a recent study reported that tumor expression of PD-L1 in melanoma is associated with the presence of TILs and a strong expression of IFNγ [23].

In another study on melanoma and non-small cell lung carcinoma (NSCLC), PD-L1 tumor expression was associated with the presence of immune infiltration. In this study, expression of PD-L1 was associated with good clinical response [24].

In addition, a recent study reported that HPV-positive head and neck squamous cell carcinoma (HNSCC) is more likely associated with intratumoral T cell infiltration, as well as PD-L1 expression, with favorable outcome [25].

Indeed, a tumor microenvironment characterized by PD-L1 expressing cells in a context of immune infiltration could be a favorable ground for immunotherapy approaches targeting regulatory immune checkpoints, such as PD-L1. Preexisting natural cytotoxic T cells at the tumor site seem to be necessary to induce anti-tumor immune response with anti PD-L1. Indeed, anti PD-L1 immunotherapy has been shown to benefit patients with preexisting T cell infiltration.

In line with such evidence, anti-PD-L1 agents have demonstrated strong clinical activity in a wide variety of tumors and are currently tested in several tumor settings [26].

In particular, a combination therapy based on Durvalumab (monoclonal antibody against PD-L1) and Tremelimumab (monoclonal antibody against CTLA-4) is currently evaluated in patients with advanced HCC (ClinicalTrials.gov Identifier: NCT02821754).

Overall, the study performed by Gabrielson and colleagues not only supports the application of the Immunoscore as prognostic marker for HCC, but also sheds light on a complex and contrasting topic that is the rationale for using PD-L1 expression as marker of prognostic significance in HCC.

## Abbreviations

AFP: Alpha-fetoprotein
AJCC: American Joint Committee on Cancer
CAFs: Cancer-associated fibroblasts
CRC: Colorectal cancer
CSLCs: Cancer stem-like cells
CTLA-4: Cytotoxic T-lymphocyte antigen 4
DKK1: Dickkopf-1
ER: Estrogen receptor
HBV: Hepatitis B virus
HCC: Hepatocellular carcinoma
HCV: Hepatitis C virus
HNSCC: Head and neck squamous cell carcinoma
HPV: Human papillomavirus
HSCs: Hepatic stellate cells
IFNγ: Interferon gamma
IGF-1: Insulin-like growth factor-1
IHC: Immunohistochemistry
Im: Immunoscore
IM: Invasive margin
NSCLC: Non-small cell lung carcinoma
OPN: Osteopontin
OS: Overall survival
PD-L1: Programmed death – ligand 1
RF: Radiofrequency
RFS: Relapse free survival
TACE: Trans-arterial chemoembolization
TAMs: Tumor associated macrophages
TI: Tumor interior
TILs: Tumor-infiltrating lymphocytes
TNM: Tumor, nodes, metastasis classification
Tregs: T regulatory cells
UICC: Union for International Cancer Control
VEGF: Vascular endothelial growth factor
